# Evolution of natural sea surface films: a new quantification formalism based on multidimensional space vector

**DOI:** 10.1007/s11356-017-0788-2

**Published:** 2017-12-03

**Authors:** Katarzyna Boniewicz-Szmyt, Stanisław Józef Pogorzelski

**Affiliations:** 1 Department of Physics, Gdynia Maritime University, 81-225 Gdynia, Poland; 2 Institute of Experimental Physics, Faculty of Mathematics, Physics and Informatics, University of Gdansk, 80-308 Gdansk, Poland

**Keywords:** Natural surfactant films, Adsorptive-viscoelastic rheology, 2D thermodynamics, Dimensionless structure vector, Cartesian distance classification, Pollution monitoring

## Abstract

Spatial and temporal variability of natural surfactant sea surface film structural parameters were evaluated from force-area isotherms, film pressure-temperature isochors, dynamic surface tension-time relations performed on samples collected in Baltic Sea shallow coastal waters. The film structure state was postulated as a 10-D dimensionless vector created from the normalized thermodynamic, adsorptive, and viscoelastic film parameters. The normalization procedure is based on the concept of self-corresponding states known in thermodynamics. The values taken by all the reduced parameters indicated a significant deviation from the reference ideal-2D gas behavior. The exhibited deviations of the surface parameters from the background values of the same thermodynamic state of each film were independent on the film-collecting procedure, sample solvent treatment, and temperature. The structural similarity was expressed quantitatively as a (Cartesian, street, and Czebyszew) distance between two vectors of the analyzed film and the standard one from the database, and appeared to be related to environmental conditions, surface-active organic matter production, and migration in the studied coastal sea region. The most distinctive parameters differing the films were *y, M*_*w*_ and *E*_isoth_, as established from Czebyszew function application. The proposed formalism is of universal concern and could be applied to any natural water surfactant system (seawater, inland water, rain water, and snowmelt water).

## Introduction

The sea surface microlayer (SML) is the thin surface layer of the ocean at the ocean-atmosphere interface that has distinctive physical, chemical, and biological properties compared to the underlying water (Zhang et al. 2003). Many constituents of the SML occur at higher concentrations than in the underlying waters (Cunliffe et al. 2013). The organic film-forming substances were considered either as dry or wet surfactants (Gladyshev 2002). Dry surfactants, such as lipids, are assumed to form the top layer above a protein-polysaccharide layer of the so-called wet surfactants. Sieburth (1983) hypothesized that the SML is a hydrated gelatinous layer formed by a complex structure of polysaccharides, proteins, and lipids, rather than more classical model of organized layers of “dry” and “wet” surfactants. Recently, it has been evidenced that SML has a gel-like nature of varying thickness (20–150 μm, Cunliffe et al. 2013) with dissolved polymeric carbohydrates and aminoacids present as well as gel particles (Cunliffe and Murrell 2009), such as transparent exopolymer particles (TEP) of polysaccharidic composition and Coomassie stainable particles (CSPs) of proteinaceous composition. Consequently, the SML phenomenon demonstrates a complex time-dependent interfacial system that requires several physicochemical, thermodynamic, elastic quantities to be considered together to figure out its evolution with environmental factors.

From a methodological point of view, numerous physical, chemical, and other methods are available for measurements of sea surface natural surfactant film structure evolution, although they can be not cost-effective, sensitive enough and practical in oceanographic investigations. It is of particular interest to make near real-time and on-site measurements of microlayer film signatures using a probing technique that responds to a broad class of organic film-forming components, and that provides specific quantitative information on a thermo-elastically complex, highly heterogeneous interfacial system with internal transition processes of different time scale and origin. Such an experimental film collection technique, theoretical background of interfacial data processing, and finally comprehensive film signatures classification stand for a complete oceanographic procedure presented here, and tested in a coastal area of the Baltic Sea (Gulf of Gdansk, Poland).

The novel classification (quantification) method of natural film structure presented here, based on physical attributes, required to specify a group of the sensitive film parameters originating from commonly available the film surface pressure-area isotherms, surface pressure-temperature isochors, dynamic surface pressure-time plots (Pogorzelski and Kogut 2001b), and stress-relaxation dependences (Pogorzelski et al. 2006). The static and dynamic structural parameters reflected the natural film morphology and resulted from the generalized physical formalisms adopted to multicomponent surfactant films of generally undetermined chemical composition.

The selected static film structural parameters turned out to reflect in a sensitive and quantitative way the film physicochemical composition (*A*_lim_, *M*_W_, *E*_isoth_), surface material concentration (*π*_*s*_, *Г*_*s*_), film molecules mobility, their interfacial interaction strength and thermal properties (*β*_*s*_, *π*_*k*_), film material solubility(*R*, *ΔS*_*c*_), 3D interfacial architecture, and miscibility of its film-forming components (*y*) (Mazurek et al. 2008). The adsorption kinetics parameters were the effective diffusion coefficient (*D*_eff_ */D*_mon_), related to the 2D film molecule aggregation number (~ *N*_aggr_ *= D*_mon_ */D*_eff_), and activation energy barrier *E*_*a*_ */R*_*g*_ *T* are both attributed to mixed adsorption mechanism at the air/water interface met in natural sea layers (Pogorzelski and Kogut 2001b; Pogorzelski et al. 2006). The formalisms presented here do not require the film surfactant concentrations and their physicochemical identification to be determined.

The normalization procedure provides deviations of the surface parameters from the reference values corresponding to the same 2D thermodynamic state of each film, which is not affected by the film-collecting procedure, possible film material solvent extraction, sample temperature, or other environmental conditions. It should be pointed out that the novel submersible film-collecting vessel was applied in these film studies where an undisturbed sea area region is “cut out”, and no any film material transfer or chemical sample processing takes place in order to further perform Langmuir trough measurements (Pogorzelski 1992; Pogorzelski et al. 1994).

A set of the normalized, dimensionless structural parameters are presented as coordinates of a multidimensional vector quantifying the film structure state. Such a concept was originally suggested for the first time in Mazurek et al. 2008, although its experimental verification required the large-scale field experiment to be performed in the Baltic, as reported here. In brief, from a classification point of view, one can treat the film structure state as a sequence of numbers—a vector in a 10D space (Borg and Groenen 2005). The structural dissimilarity between two films in question can be expressed quantitatively as a distance (metrics) between two vectors of the analyzed film and the reference one from the collected database, respectively. In future studies, a simple Cartesian metrics can be replaced as a comparing measure may turn out insufficient, and we will have to replace with the more sophisticated routine (introducing weighting functions related to the particular parameter, for example) (Mazurek et al. 2008).

The aim of the study was to demonstrate that a multidimensional structure vector classification approach of the natural sea surface film thermo-elastic and physicochemical state is a promising tool for its spatial and temporal evolution monitoring with solely physical attributes. It could be developed to an alternative, low-cost, automatic technique useful in oceanographic pollution assessment.

## Theoretical principles: surface parameters as sensitive indicators of natural film features

Sea surface film-forming natural surfactants create 2D interfacial structures which complete compositional and structural description is not currently feasible. As postulated by the authors, it should be possible to scale microlayer film surface pressure-area isotherms and surface pressure-temperature isochors in terms of the structural parameters, reflecting the natural film morphology, 2D thermodynamics, viscoelasticity, and adsorptive features and resulting from the generalized physical formalisms adopted to multicomponent surfactant films, as already demonstrated in (Pogorzelski 2001; Pogorzelski and Kogut 2001a, 2001b, 2003a, 2003b). They are quoted here together with the properly controlled experimental conditions required to obtain the reliable film parameters, and stand for an essential interpretational background.

## Static film structure parameters

The isotherms *(π-A)*_*T*_, for natural films, differ significantly from ideal 2D gas behavior *πA*_*m*_ *=* kT, that is why the following quadratic equation of state, e.g., the 2D virial equation was proposed and scaled according to specific area (Barger and Means 1985; Frew and Nelson 1992):

1 $$ \pi A={C}_0+{C}_1\pi +{C}_2{\pi}^2, $$

where

*π = γ*_0_-*γ*: is the film surface pressure,
*γ*_0_, *γ*: the surface tensions of solvent (water) and surfactant solution, respectively,
*A*_*m*_: the area per film molecule related to the Gibbs’ adsorption *Г*; *A*_*m*_ *= 1/ГN*_*A*_,
*k*: the Boltzmann constant,
*T*: the temperature in Kelvins,
*N*_*A*_: the Avogadro number,
*C*_0_, *C*_1_, *C*_2_: are the virial coefficients,
*A*: is the film-covered area (in cm^2^)

The virial coefficients, resulting from the best-fitting procedure applied to the registered isotherms *(π-A)*_*T*_, can be related to the mean number of moles *n*_*m*_ present in the film, specific limiting area *A*_lim_, and mean molecular mass *M*_*w*_ of the film-forming surfactant mixture, as described in detail elsewhere (Pogorzelski 2001; Pogorzelski and Kogut 2001a, 2003a).

The dilational elasticity modulus *E*_isoth_ exerting the static, compressional response of a film to compression-dilation surface area deformation taken at the isotherm registration in its thermodynamic equilibrium (Adamson 1982):

2 $$ {E}_{\mathrm{isoth}}=-\frac{d\pi}{d\ln A}, $$

can be used to distinguish the 2D film states in marine films (Pogorzelski and Kogut 2003a). Enthalpy *∆H* and entropy *∆S*_*t*_ of the first-order phase transitions (G → LE → LC) were evaluated using the Clausius-Clapeyron equation applied to the force-area isotherms taken at different temperatures (Pogorzelski 2001).

The isotherm hysteresis is a phenomenon reflecting different arrangements of the film-forming molecules in the closed compression-expansion cycles, and is attributed to a structural entropy change *∆S*_*c*_ *(∆S*_*c*_ *= ∆W/T*, where *∆W = W*_dil_ *− W*_com_ is the difference in the work derived from the integration routine applied to the expansion and compression isotherm plots) of the interfacial system.

The isotherm reversibility *R*:

3 $$ R=100\times \left(\frac{W_{\mathrm{dil}}}{W_{\mathrm{com}}}\right)\%, $$

stands for a qualitative measure of the entropy effect of surface films differing in their chemical structure depending on the film deformation velocity (Hűhnerfuss and Alpers 1983), and evidently related to solubility of the film-composing material, as shown for natural seawater samples (Pogorzelski et al. 1994).

2D phase separation of the monolayer-forming components in a heterogeneous film can be considered in the framework of the 2D polymer film-scaling theory (De Gennes 1979; Jiang and Chiew 1994). The scaling exponent (*y*)—can be obtained from the relation *E*_isoth_ *= yπ*, by measuring the high-frequency limit of the surface modulus *E*_isoth_ as a function of the surface pressure (Pogorzelski 1996). For low values of *y* (< 3.5), the interfacial film-water subphase system demonstrates a “good” solvent behavior and one is concerned with a homogeneous monomolecular mixed film. Higher *y* values (*y* ≈ 8) lead to less film homogeneity observed as patches or domains (2D micelles) of film-forming components. For highest *y* values (> 10–16), the system indicates the vertical segregated film structures forming nearly separate horizontally situated layers at the interface (sandwich-like) where the most insoluble (hydrophobic) compound is placed on the outermost surface of this layered system (Mann et al. 1993).

Intermolecular interactions between the film-forming components can be obtained from 2D thermodynamics studies (Defay et al. 1977). In particular, the surface pressure-temperature isochore *(π-T*)_*A*_. dependence which leads to the surface pressure-temperature coefficient *β*_*s*_ *= (∂π/∂T)*_*A*_ (also called the surface entropy *S*_*s*_ = *(∂γ/∂T)* considered recently in (Boniewicz and Pogorzelski 2016) is related to the thermal translational molecules motion quantified with the kinetic surface pressure *π*_*k*_ *= β*_*s*_ *T* (Rosenholm et al. 2003). The isoarea temperature *β(T)* dependence demonstrates the inflection points indicating the presence of particular critical phenomena (partial film collapse, 2D–phase transitions of higher orders, compression-induced structural conformation changes) not reflected in surface pressure-area isotherms (Rosenholm et al. 2003). The resulting surface pressure of the film can compose with the following components: *π = π*_*k*_ *+ π*_*c*_ *+ π*_*r*_, where *π*_*c*_ stands for the cohesive surface pressure attributed to the van der Waals forces of attraction between the hydrocarbon chains, and *π*_*r*_ is results from the electrostatic forces of repulsion between charged head groups of monolayers (Gong et al. 2002). The latter term appears to be negligible for surfactants of neutral character (nonionic ones). For long chain surfactants *π*_*c*_ ≈ − 400 *m*_*m*_ *A*_*m*_^−3/2^, for *A*_*m*_ > 1 nm^2^, where *m*_*m*_ denotes the number of methylene groups in the hydrocarbon chain and *A*_*m*_ is expressed in Ǻ^2^ as demonstrated in Davies and Rideal 1961. *π*_*c*_ is a sensitive measure of the subphase pH effect on ionic surfactant molecules interactions (Gong et al. 2002; Miranda et al. 1998).

The reliable film parameters can be obtained only under certain and properly controlled experimental conditions. In order to fulfill such requirements, the film parameters (*n*_*m*_ *, A*_lim_ *, M*_*w*_) are derived from the virial coefficients obtained via the best-fit procedure (a least-squares fitting algorithm with a significance level 0.95) applied to the registered *(π-A)*_*T*_ isotherms within the initial surface pressure interval 0–2 mN m^−1^ where a surface film demonstrates the ideal 2D gas behavior. The elasticity modulus *E*_isoth_ is computed from the initial part of the isotherm (0 < *π* < 1 mN m^−1^) according to Eq. (2). Further parameters *∆S*_*c*_ and *R* result from the integration routine of the compression and expansion isotherm plots. The scaling exponent y is determined from the relation *E*_isoth_ *= yπ* from its low surface pressure range (0 < *π* < 2–3 mN m^−1^) part, as shown in Pogorzelski 1996. The surface pressure temperature coefficient *β*_*s*_ is obtained from a slope of the straight line tangent to the experimental plot computed using a least-squares fitting procedure (values of *r* ^*2*^ were ranging from 0.83 to 0.96; where *r* is the correlation coefficient), and applied to the particular temperature ranges below and above the cusp points evidenced at each isochore plots (see Fig. 3 in Mazurek et al. 2006; Mazurek et al. 2006a). Additional particulars on the determination procedure the film structural parameters from the measured isotherm *(π-A)*_*T*_ and isochore *(π-T)*_*A*_ plots are given elsewhere (section 3 in (Mazurek et al. 2006)).

The thermodynamic equilibrium in the film is a serious problem in the light of the phase transitions evidenced therein. The effect can be quantified by means of the dimensionless parameter Deborah (*De*) number defined as the ratio of the film relaxation process time *τ* to the *t*_obs_ *—*“time of observation” (a reciprocal of the strain rate of a film: *t*_obs_ *=* [(*dA/A*)*/dt*]^*−*1^), as discussed in (Kato et al. 1992). At sufficiently low film area compression velocities (= *ΔA/Δt*), *De* parameter is less than unity, and the film system is in its quasi-equilibrium thermodynamic state. Any relaxation process in films leads to surface viscoelasticity and may affect the shape of isotherms and consequently the recovered film parameters (Jayalakshmi et al. 1995). Several real interfacial surfactant systems are visco-elastic, and the dilational modulus is a complex quantity composed of real *E*_*d*_ (dilational elasticity) and imaginary *E*_*i*_ (=*ωη*_*d*_, where *η*_*d*_ is the surface dilational viscosity and *ω*—the angular frequency of periodic surface area oscillations) parts: *E = E*_*d*_ *+ iE*_*i*_. The dilational viscoelasticity modulus can be approximated by its static Gibbs analogue *E*_isoth_, under low film area deformation rates (*De* < <1).

## Dynamic film parameters

To determine 2D viscoelasticity of natural surfactant films and the characteristic times of transition processes resulted from surface deformation stress, the method proposed in (Joos et al. 1992; Serrien et al. 1992) was used. The step and rapid (*∆t*~0.1–0.7 s) relative area deformation (*∆A/A*_0_ = 0.1–0.3) was applied to the film, and the surface pressure-time decay curve was analyzed, as described in Pogorzelski and Kogut 2001a. The surface rheokinetic parameters, collected in Table 6 of Boniewicz and Pogorzelski 2016, demonstrate that we are concerned with elastic films (*E*_*d*_ » *E*_*i*_). Recent natural seawater film studies revealed a two-step relaxation process with characteristic times *τ*_1_ (1.1–2.8) and *τ*_2_ (5.6–25.6) seconds (Boniewicz and Pogorzelski 2016).

The novel approach proposed for the description of surfactant adsorption kinetics is based on the mixed kinetic-diffusion model (Eastoe et al. 2001). The adsorption kinetics parameters—the effective relative diffusion coefficient *D*_eff_ */D*_mon_ and activation energy barrier *E*_*a*_ */R*_*g*_ *T* are obtained from a slope of the dynamic surface pressure *π(t)* dependences at short (*t*→0) and long (*t*→∞) adsorption time intervals, where the saturation film pressure *π*_*s*_, and Gibbs adsorption *Γ*_*s*_ *=π*_*s*_ */R*_*g*_ *T* are supplementary entering quantities (*R*_*g*_ is the gas constant, and *D*_mon_, the monomer diffusion coefficient (Pogorzelski et al. 2006)). According to the classical Stokes-Einstein formula, the monomer diffusion coefficient *D*_mon_ = *kT*/6*πηR*_mon_, where *η* is solution viscosity, and *R*_mon_ is the surfactant molecule monomer radius (Birdi 1997). It has been shown (Kragel et al. 1995), for natural surfactant films extracted from seawater (Tyrrhenian Sea, Italy), that the majority of biopolymeric molecules are probably aggregated as *D*_eff_ < < *D*_mon_. The adsorption kinetics parameters are used to evaluate surfactants adsorption ability (surface activity) and 2D interfacial molecular aggregation number *N*_aggr_ (Pogorzelski et al. 2006). It appears that the radius of surfactant aggregates *R*_aggr_ » *N*_aggr_ *R*_mon_, and the aggregation number *N*_aggr_ can be expressed by *D*_mon_/*D*_eff_. For the recently studied Baltic Sea microlayer samples, *N*_aggr_ values were ranging from 6.8 to 125.0 (Boniewicz et al. 2017; manuscript in preparation).

## Film structure multidimensional vector approach

A normalization concept originates from the theory of system thermodynamic states corresponding to each other (Cengel and Boles 2007; Woodcock 2016). The film state equation can be expressed in a reduced form, introducing the parameters normalized to the values taken at a particular condition (here mostly at the gaseous state but also at the critical state as applied in physical chemistry of 3D liquids and gases). Such an equation consists of only dimensionless number quantities. As a consequence, two interfacial films with a set of the same normalized surface parameters represent the same thermodynamic state. The variability of the reduced-normalized parameters introduced here rather than their absolute values is postulated to be a useful measure of the film structural state and a tool for natural water surfactant pathways tracing and reflecting the film spatial, temporal, and seasonal signatures. If values taken by the reduced parameters are > 1, that indicates a significant deviation of the particular film from the reference ideal-2D gas behavior. The normalization approach reflects deviations of the surface parameters from the background values corresponding to the same thermodynamic state of each film, independent on the film-collecting procedure, solvent sample treatment, or sample temperature. That brings out the effect of film chemical composition diversity, source-specific component, and film morphology evolution with environmental factors on the film signatures making it more accessible from the parameters data set. However, for the first time, the apparent solvent polarity effect on the structure and surface parameters of ex-situ-formed natural sea microlayer films was already quantitatively evaluated by means of the author’s scaling procedures applied to unique Brewster angle microscopy and Langmuir trough isotherm data on Mediterranean Sea samples obtained by others (Mazurek et al. 2008). It would be of interest to perform the same analysis approach on sea-surface layer sampled with different film-collecting devices (Garrett screen etc.).

In order to create the multidimensional film structure vector, first the experimentally derived (*π-A*)_*T*_, (*π-T*)_*A*_, and *π*(*t*) dependences have to be evaluated to get the normalization base parameters. All of them considered in our practice are collected in Table 1. There are also well known parameters already determined in oceanographic film characterization studies (*M*_*w*_(C_17_), *Γ*_*∞*_; Liss 1975; Hunter and Liss 1981), and taken from classical 2D thermodynamics (*R*_g_, *kT*, *dγ/dT*; Birdi 1997).

**Table 1:** Sea surface film structure parameters and their normalized values

No.	Parameters	Basis of normalization	Normalized parameter	Remarks
1	*M*_*w*_	*M*_*w*_(C_17_) = 282 Da	${\tilde{M}}_w$ = *M*_*w*_/*M*_*w*_(C_17_)	*M*_*w*_(C_17_) molecular mass of long-chain lipids≈282 Da (Liss 1975)
2	*A*_lim_	*A*_gas_ = kT*/π*_lim_	${\tilde{A}}_{\mathrm{lim}}$ = *A*_lim_/*A*_gas_	*A*_gas_ film molecular area at gaseous state
3	*E*_isoth_	*π*_lim_	${\tilde{E}}_{\mathrm{isoth}}$ = *E*_isoth_/*π*_lim_	= 1 for gaseous state
4	*R*	–	$\tilde{R}$ = *R*	No normalization
5	*ΔS*_*c*_	*C*_0_/*T*	$\Delta {\tilde{S}}_c$ = *ΔS*_*c*_/0.5 nk	0.5*nk* thermal motion entropy of 2D ideal gas
6	*y*	–	$\tilde{y}$ = *y*	No normalization
7	*ΔS*_*t*_	*C*_0_/T	$\Delta {\tilde{S}}_c$ = *ΔS*_*c*_/0.5 nk	As in 5
8	*ΔH*_*t*_	*C*_0_ = 0.5 nkT	$\Delta {\tilde{H}}_t$ = *ΔH*_*t*_/*C*_0_	*C*_0_ thermal motion enthalpy of 2D ideal gas
9	*β*_*s*_	dγ/dT = − 0.15 × 10^−3^mN m^−1^ K^−1^	${\tilde{\beta}}_s$ = *β*_*s*_/(−0.15 × 10^−3^)	dγ/dT surface tension temperature coefficient of water at 20 °C
10	*π*_*k*_	*π*_lim_	${\tilde{\pi}}_k$ = *π*_*k*_/*π*_lim_	*π*_lim_ limiting film pressure corresponding to *A*_lim_
11	*Γ*_*s*_ = *π*_*s*_/*R*_*g*_ *T*	*Γ*_*∞*_ = 8 × 10^−9^ kmol m^−2^	${\tilde{\Gamma}}_s$ = *Γ*_*s*_/*Γ*_*∞*_	*Γ*_*∞*_ saturation adsorption for long-chain (mean CH_2_ group number = 16.8) lipids
12	*π*_*s*_	*π*_lim_	${\tilde{\pi}}_s$ = *π*_*s*_/*π*_lim_	as in 10
13	*D*_eff_	*D*_mon_	${\tilde{D}}_{\mathrm{eff}}$=*D*_eff_/*D*_mon_	*D*_mon_ monomer diffusion coefficient
14	*E*_*a*_	*R*_*g*_ *T*	${\tilde{E}}_a$ = E_a_/*R*_*g*_ *T*	

A dimension of the film structure vector can be rationalized by excluding the parameters closely correlated to each other since they born no more information on the film properties. A further step is to select a functional form of the distance in a multidimensional space. Exemplary dependences commonly used in multidimensional analyses are (Borg and Groenen 2005) the following:

1. Cartesian—geometric distance in a *n*-dimensional space between vectors “*i*” and “*j*” consisting from *x*_*k*_ components (from *k* = 1 to *n*):

4 $$ \left({d}_{\mathrm{Cartesian}}\right)={d}_{\mathrm{ij}}=\sqrt{\sum \limits_{k=1}^n{\left({x}_{\mathrm{ik}}-{x}_{\mathrm{jk}}\right)}^2}. $$

2. Street distance:

5 $$ \left({d}_{\mathrm{street}}\right)={d}_{\mathrm{ij}}=\sum \limits_{k=1}^n\left|{x}_{\mathrm{ik}}-{x}_{\mathrm{jk}}\right|. $$

3. Czebyszew distance suitable for establishing the difference in one particular parameter (dimension):

6 $$ \left({d}_{\mathrm{Czebyszew}}\right)={d}_{\mathrm{ij}}=\max \left|{x}_{\mathrm{ik}}-{x}_{\mathrm{jk}}\right|,\kern0.5em k=1.\dots n $$

## Experimental methodology

### Sampling sites

Natural marine surfactant film surface rheology and adsorption studies in shallow off-shore waters of the Baltic Sea (Gulf of Gdańsk, Poland) were carried out in April–May, 2013 at selected locations along the coast from Brzeźno to Gdynia (shown in Fig. 1 of Pogorzelski et al. 2006).

**Fig. 1:**
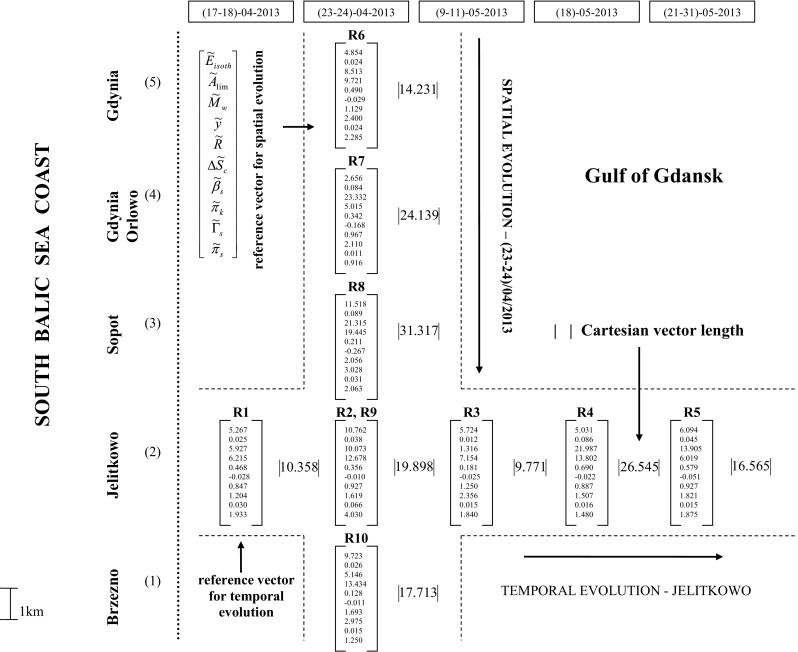
Film structure vector spatial and temporal evolution in Baltic Sea coastal waters. For denotations see text. Boniewicz-Szmyt and Pogorzelski, Evolution of natural sea surface films: a new quantification formalism based on multidimensional space vector

Sea surface microlayer samples were collected mostly under calm sea conditions (wind speed at a10-m height *V*_*10*_ < 4 ms^−1^) from a rubber boat or a pier (platform).

### Methods

The novel film sampler is a submersible rectangular double-walled vessel which “cuts out” an undisturbed sea area region and is integrated with a conventional Langmuir trough placed therein. The most valuable advantage of this system over other film-collecting methodology is that the collection process and Langmuir trough force-area studies are performed without transferring and any chemical processing of the microlayer material, as described in Pogorzelski 1992; Pogorzelski et al. 1994. After film microlayer sampling, the trough is subsequently put on the top of the measuring table where the remaining mechanical and electronic devices of the experimental arrangement are located near the shore line (as shown in Fig. 1 of (Boniewicz and Pogorzelski 2016)). To perform surface isotherm studies, the initial Langmuir trough area *A*_0_ = (1200 cm^2^) is compressed with an average deformation speed *v* = 0.6 cm^2^ s^−1^ (corresponding to *De* value~0.09) by moving two paraffin wax-coated glass sliders towards each other symmetrically around the film pressure sensor (Wilhelmy plate technique using a piece of filter paper 5 cm wide attached to the force sensor). A detailed description of the measuring procedures and physical conditions adopted in surface pressure-area isotherm, surface pressure-*T*, and dynamic surface pressure registrations can be found in Pogorzelski and Kogut (2001a, 2001b, 2003b). For in situ seawater surfactant adsorption dynamics studies, a hand-held bubble pressure tensiometer (PocketDyne BP2100, Krüss, Germany) with an adjustable surface age was used.

## Results and discussion

In comprehensive considerations, the diagram of natural film vector distribution was used, shown in Fig. 1, constructed as a result of measurements performed within an approximately 1-month period (17–18 April, 2013 to 21–31 May, 2013) in coastal waters of the Baltic Sea along a coastal line at five stations from Brzeźno to Gdynia. A sequence of the normalized parameters, indicated with a wavy line over the symbol letter, was from *E*_isoth_ to *π*_*s*_ as summarized in the upper left-hand part of Fig. 1. The registrations R1→R5 allowed one to consider the temporal film evolution (at Jelitkowo) whereas R6→R10 records reflected the spatial parameters variability at the particular moment (23–24 April, 2013). Further parameters from a wide list collected in Table 1 can be further included that is attributed to the particular aim of the oceanographic studies, in particular, to the surface film-mediated process taking place at the air/sea interface where the surface structural, rheological, diffusional, and thermodynamic signatures play a key role.

Since the selected surface parameters reflect several mechanisms and signatures of the film structure evolution such as molecular composition (related to *A*_lim_, *M*_*w*_, *E*_isoth_), film solubility and components miscibility (via *R*, *∆S*_*c*_, and *y* factors), surface concentration (*Γ*_*s*_, *π*_*s*_), and film molecules mobility (*β*_*s*_, *π*_*k*_), the particular normalized vector components analyzed alone provide a synthetic characteristics of the film structure. On the basis of the presented data, the following film signatures can be distinguished, as evidenced from the parameters evolution on the large data set in Mazurek et al. 2008:

1. Baltic Sea films differed significantly from the 2D ideal gas (G) layers (${\tilde{E}}_{\mathrm{isoth}}$> > 1, ${\tilde{A}}_{\mathrm{lim}}$≈10^−2^) did not form saturate surfactant layers (${\tilde{\Gamma}}_s$~ 10^−2^, ${\tilde{\pi}}_s$~ 2). Similarly, polynomial approximations of the natural film isotherms applied for samples from the near shore of the Black Sea and Atlantic Ocean coastal waters exhibited significant deviations from the 2D ideal gas behavior (Averbukh et al. 2014).
2. Film-forming molecules appeared in an aggregated form like biopolymeric materials (${\tilde{M}}_w$~ 10–20, *D*_eff_ < < *D*_mon_; *D*_eff_ */D*_mon_ = 0.01–0.87) and formed non-uniform surface structures ($\tilde{y}$ = 6.0–19.4) containing surface-active components of differentiated solubility in the water phase ($\tilde{R}$ = 0.18–0.69).
3. 2D thermodynamics parameters (values of $\Delta {\tilde{H}}_t$ and $\Delta {\tilde{S}}_t$found are characteristic for the first-order gas-liquid expanded transitions observed for model lipid-formed monolayers on water (Adamson 1982), and ${\tilde{\beta}}_s$ = 0.847–2.056) turned out to be comparable to these reported for interfacial layers of tridecyclic, myristic, and pentadecyclic acids (Birdi 1997).

**Fig. 2:**
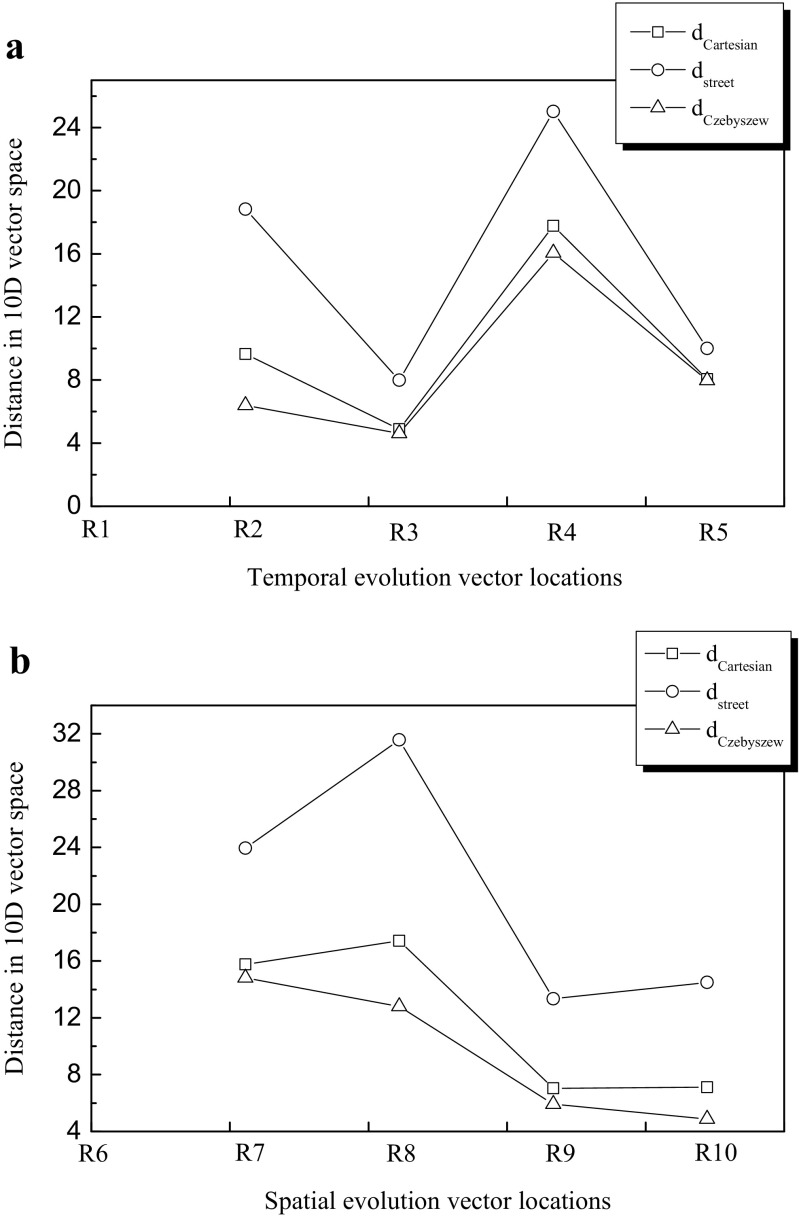
**a** Temporal and **b** spatial evolution of distance between surface film structure vectors in a multidimensional space measured with different distance functions: Cartesian, square; street, circle; and Czebyszew, triangle. Baltic Sea coastal waters case studies. R1 and R6 stand for the reference films (vectors). Boniewicz-Szmyt and Pogorzelski, Evolution of natural sea surface films: a new quantification formalism based on multidimensional space vector

Cartesian vector length, given on the right-hand side of the vector column, is a sensitive measure of the short-term changes of the film structure (compare R1→R5) registered at Jelitkowo. The largest vector lengths were observed at R2 (*d*_Cartesian_ = 19.898) and R4 (*d*_Cartesian_ = 26.546) sampling times in reference to the initial R1 (*d*_Cartesian_ = 10.358) that could be a result of the nature of hydrodynamic and atmospheric conditions further related to the surface water mixing at shallow sea regions. An increase of the vector length was observed for the spatial evolution starting from R6 (Gdynia) to R10 (Brzeźno). The highest value was noticed at Sopot (*d*_Cartesian_ = 31.317) where the municipal effluents are expected to contribute significantly in surface-active film-forming matter. Generally, *d*_Cartesian_ higher values were evidenced in locations close to town areas and the mouth of a river (R7). It is of interest to consider whether other distance functions operating in a multidimensional vector space can be equally useful or provide additional information. Temporal and spatial evolution of structure vector distance between the reference vectors R1 and R6 versus sampling locations, for different forms of the metric function (Cartesian, street, and Czebyszew), are shown in Fig. 2a, b, respectively. All the distance function forms lead to the same variability trend of the both relations *d*(R1→R5) and *d*(R6→R10) in Fig. 2a, b. However, the dynamics of the variability and the absolute distance values were highest for *d*_street_ and lowest for *d*_Czebyszew_ at all the locations and the characteristic points (maxima and minima). Czebyszew distance function allows one to point out the most distinctive parameter for the base and considered vectors (differing the both vectors in question), as it is evident from Eq. (6). From data in Fig. 1, it can be learned that the most distinctive parameters for these Baltic sea films were$\tilde{y}$, ${\tilde{M}}_w$, and ${\tilde{E}}_{\mathrm{isoth}}$. It should be noticed (see Fig. 2b lines between R9 and R10) that street, Czebyszew, and Cartesian distance functions could lead to misleading conclusions on the evolution direction since the lines are pointed up, down, or horizontal, respectively, under certain circumstances.

In a further stage of data processing, it could be possible to construct the transfer function (in a form of matrix) of the film structure evolution between vector “*i*” and “*j*” if larger comprehensive data would be collected.

$$ \left[\begin{array}{c}{x}_{i1}\\ {}{x}_{i2}\\ {}\cdot \\ {}\cdot \\ {}{x}_{in}\end{array}\right]=\left[\begin{array}{cccc}{a}_{11}& & & {a}_{1n}\\ {}& & & \\ {}& & & \\ {}& & & \\ {}{a}_{n1}& & & {a}_{nn}\end{array}\right]\times \left[\begin{array}{c}{x}_{j1}\\ {}{x}_{j2}\\ {}\cdot \\ {}\cdot \\ {}{x}_{jn}\end{array}\right] $$

In addition, the film structure quantification with a multidimensional approach allows application of multivariate statistical methods. From among many statistical techniques, principal components analysis (PCA) also known as empirical orthogonal function analysis and cluster analyses (CA) are frequently applied to geochemical data sets (Meglen 1992). In brief, PCA is commonly used on data sets which consist of columns-representing sample properties and rows representing individual samples.

It is interesting to compare the structural parameters of atmospheric water surfactant films (on rainwater and snowmelt samples) studied in neighboring sea (Gulf of Gdańsk, Baltic Sea) coastal areas (Mazurek et al. 2006, 2006a). It appears that the surface-active material of snow water is less soluble (higher *R*) of similar chemical nature (comparable *E*_isoth_ and *y* values) if compared to the surfactant films evidenced in rainwater. In fact, the novel film structure classification approach seems to be of general concern applicable to a variety of natural water surfactant systems.

It should be borne in mind that the surface film parameters exhibit spatial-temporal-seasonal evolution, and are related to the particular biological event (s) features. To support such an assumption, the following examples can be given. It could be learned from monolayer studies and a force-area quantification approach applied for physicochemical characterization of surface-active substances of the sea surface microlayer from Middle Adriatic stations (Frka et al. 2012). Higher primary production during late spring-early autumn was reflected in the appearance of films of higher surface activity containing compounds of lower molecular masses (*M*_*w*_ = 0.65 ± 0.27 kDa) and higher miscibility (*y* = 6.46 ± 1.33), and elasticity modulus (*E*_isoth_ = 18.35 ± 2.02 mN m^−1^) in comparison to structural parameters (*M*_*w*_ = 2.15 ± 1.58 kDa; *y* = 3.51 ± 1.41; *E*_isoth_ = 6.41 ± 1.97 mN m^−1^) obtained for samples collected in a period of lower biological production (Frka et al. 2012). Force-area studies performed on chlorophyll-a surfactants at the air-water interface revealed the following values of *y* = 5.3–8.2, *A*_lim_ = 59–67 Ǻ^2^ molec ^−1^, *E*_isoth_ = 69–95 mN m^−1^ and exhibited 2D solid-like behavior (Periasamy 2012). The effect of nanogel colloidal and dissolved organic matter < 0.2 μm (Fuentes et al. 2011), secreted by marine biota on surface properties of films spread at air-water interface, was already studied by means of the same scaling approach as presented in our studies. The isotherms presented therein are typical for surface-active compounds forming expanded films with similar characteristics (*y* = 4.2–6.2; *E*_isoth_ = 14–26 mN m^−1^, *A*_lim_ = 171.2–322.4 Ǻ^2^ molec.^−1^, *M*_*w*_ = 1.37–2.37 kDa) as evidenced in experiments with marine surfactant films (Mazurek et al. 2008).

High values of *M*_*w*_ provide experimental evidence of the existence of truly colloidal substance in the organic pool isolated from the seawater samples (Thornton 2014; Thornton et al. 2016). These values remain in agreement with *M*_*w*_ of the colloidal nanogels reported in the literature (Verdugo et al. 2004). Transparent exopolymer particles (TEP) are surface-active macro gels that play a role in the marine carbon cycle by spanning the size continuum between dissolved and particulate organic carbon, in addition to supporting particle aggregation (Jennings et al. 2017). The enrichment of transparent exopolymer particles in the microlayer and the subsequent production of a gelatinous biofilm have implications on air-sea gas transfer and the partitioning of organic carbon in surface waters (Cuncliffe et al. 2011). Micro gel aggregation rates in SML have been shown to be fivefold higher than in underlying water just a few tens of cm below the SML (Wurl et al. 2017; Taylor et al. 2014). Gel particles can promote microbial biofilm formation (Bar-Zeev et al. 2012) and mediate vertical organic matter transport. Accumulation of organic matter in SML may be closely coupled to phytoplankton abundance in the water column (Galgani et al. 2014). So, organic matter accumulation and composition in SML may also reflect the sensitivity of marine organisms in the surface ocean to environmental changes which was shown in mesocosms studies (Stolle et al. 2011).

In “real” systems, in technology, biology, and oceanography, surfaces are often non-uniform. For instance, a flat surface containing a surfactant monolayer which has undergone a two-dimensional phase separation falls under this definition, as well as air-water and oil-water interfaces with droplets, solid particles, or even thin layers of a microemulsion, foam, or a bicontinuous phase of complex surface thermo and viscoelastic properties (Maestro et al. 2015; Maestro et al. 2014; Mazurek and Pogorzelski 2012). Addition of surface-active component to seawater can lead to complex formation and affect the interfacial properties (Guzman et al. 2014). Further undetermined components of SML are micro-sized synthetic polymer particles capable of accumulating at the air-water interface (Song et al. 2014), consisted of polypropylene, polyethylene, phenoxy resin, polystyrene, polyester, synthetic rubber, and other polymers are likely to be present in coastal sea zones (Song et al. 2015).

It is of interest to clarify the role of nitrogen-containing surfactants in controlling film elasticity *E*_isoth_ (Bock and Frew 1993). The enchanced contributions of relatively soluble biopolymeric components such as proteins in the sea surface microlayer would be reflected in lower C/N ratio values. It should be noted that the relation *E*_isoth_ as a function of C/N was already derived with a high correlation coefficient value (Pogorzelski et al. 2006). So, higher C/N ratios were accompanied with higher *E*_isoth_ values, pointing to incorporation of nitrogen-rich compounds or other biopolymeric materials that lead to lower film elasticity (Pogorzelski et al. 2006).

## Conclusions and future work

Natural surfactant films exhibit the multicomponent character being a mixture of biopolymeric molecules covering a wide range of solubilities, surface activity, and molecular masses demonstrating a spatial-temporal-seasonal variability which can be quantified with several surface rheology parameters. Certain classes of components or “end-members” are slowly degraded or are transformed to even more stable chemical structures and thus can be used as source-specific surface-active biomarkers to trace environment state ecological changes. The presented approach based on the physical states similarity theory provides a universal measure of surface film structure evolution independent on the film-collecting procedure, solvent treatment, and sample temperature applicable to a wide variety of the original films met in natural waters (marine, inland, and atmospheric). The variability of the reduced-normalized parameters introduced here rather than their absolute values is postulated to be a useful indicator of the film structural state, and can be used as a tool for natural water surfactant pathways tracing and reflecting the film spatial, temporal, and seasonal signatures.

A set of the normalized structural parameters can be treated as coordinates of a multidimensional vector in a 10D space quantifying the film structural state. The structural similarity of the considered films can be expressed quantitatively as Cartesian distance between two vectors of the analyzed film and the standard one from the database, respectively. Application of different distance functions, i.e., street or Czebyszew may result in a better data presentation dynamics or pointing to the most distinctive parameters differing the studied films structure.

Quantification of film structure with a multidimensional vector approach allows application of Principal Component Analysis (PCA) and Cluster Analysis (CA) to the data set amended with environmental characteristics.

It is a promising starting point to create cost-effective, automatic, and rather simple technique to monitoring and assessment of environmental pollution in oceanographic practice.

## References

[CR1] Adamson AW (1982). Physical chemistry of surfaces.

[CR2] Averbukh EL, Talipova TG, Kurkin AA, Soomere T (2014). Statistical characteristics of coefficients of a cubic approximation of isotherms of surface active substance films. Proc. Estonian Acad Sci.

[CR3] Barger WR, Means JC, Sigleo AC, Hattori A (1985). Clues to the structure of marine organic material from the study of physical properties of surface films. Marine and Estuarine Chemistry.

[CR4] Bar-Zeev E, Berman-Frank I, Girshevitz O, Berman T (2012). Revised paradigm of aquatic biofilm formation facilitated by micro-gel transparent exopolymer particles. P Natl Acad Sci USA.

[CR5] Birdi S (1997). Handbook of surface and colloid chemistry.

[CR6] Bock EJ, Frew NM (1993). Static and dynamic response of natural multicomponent oceanic surface films to compression and dilation: laboratory and field observations. J Geophys Res.

[CR7] Boniewicz-Szmyt K, Pogorzelski SJ (2016). Thermoelastic surface properties of seawater in coastal areas of the Baltic Sea. Oceanologia.

[CR8] Boniewicz-Szmyt K, Pogorzelski SJ, Grzegorczyk M (2017) A gelatinous nature of the sea surface microlayer (SML) as evidenced with its thermo-viscoelastic and adsorptive surface properties (manuscript in preparation)

[CR9] Borg I, Groenen P (2005). Modern multidimensional scaling: theory and applications.

[CR10] Cengel YA, Boles MA (2007) Thermodynamics: an engineering approach. McGraw Hill, 6th edition, New York

[CR11] Cunliffe M, Murrell JC (2009). The sea-surface microlayer is a gelatinous biofilm. ISME J.

[CR12] Cunliffe M, Upstill-Goddard RC, Murrell JC (2011). Microbiology of aquatic surface microlayers. FEMS Microbiol Rev.

[CR13] Cunliffe M, Engel A, Frka S, Gasparovic B, Guitart C, Murrell JC, Salter M, Stolle C, Upstill-Goddard R, Wurl O (2013). Sea surface microlayers: a unified physicochemical and biological perspective of the air-ocean interface. Progr Oceanogr.

[CR14] Davies JT, Rideal EK (1961). Interfacial phenomena.

[CR15] De Gennes PG (1979). Scaling concepts in polymer physics.

[CR16] Defay RI, Prigogine I, Sanfeld A, Kerker MA, Zettlemoyer C, Rowell RL (1977). Surface Thermodynamics. Colloid and Interface Science 1.

[CR17] Eastoe J, Rankin A, Wat R (2001). Surfactant desorption dynamics. Int Rev Phys Chem.

[CR18] Frew NM, Nelson RK (1992). Scaling of marine microlayer film surface pressure-area isotherms using chemical attributes. J Geophys Res.

[CR19] Frka S, Pogorzelski SJ, Kozarac Z, Cosovic B (2012). Physicochemical signatures of natural sea films from middle Adriatic stations. J Phys Chem A.

[CR20] Fuentes E, Coe H, Green D, Mc Figgans G (2011). On the impacts of phytoplankton-derived organic matter on the properties of the primary marine aerosol- part 2: composition, hygroscopicity and cloud condensation activity. Atmos Chem Phys.

[CR21] Galgani L, Stolle C, Endres S, Schulz KG, Engel A (2014). Effects of ocean acidification on the biogenic composition of the sea-surface microlayer: results from mesocosm study. J Geophys Res Oceans.

[CR22] Gladyshev MI (2002). Biophysics of the surface microlayer of aquatic ecosystems.

[CR23] Gong K, Feng S-S, Go ML, Soew PH (2002). Effects of pH on the stability and compressibility of DPPC/cholesterol monolayers at the air-water interface. Colloids and Surfaces A: Physicochem Eng Aspects.

[CR24] Guzman E, Santini E, Benedetti A, Ravera F, Ferrari M, Liggieri L (2014). Surfactant induced complex formation and their effects on the interfacial properties of seawater. Colloids Surf B: Biointerfaces.

[CR25] Hűhnerfuss H, Alpers W (1983). Molecular aspects of the system water/monomolecular surface film and the occurrence of a new anomalous dispersion regime at 1.43 GHz. J Phys Chem.

[CR26] Hunter KA, Liss PS (1981) Organic sea surface films. In: Marine organic chemistry, Duursma EK and Dawson R (Eds). Elsevier, New York, pp 259–298

[CR27] Jayalakshmi Y, Ozanne L, Langevin D (1995). Viscoelasticity of surfactant monolayers. J Colloid Interface Sci.

[CR28] Jennings MK, Passow U, Wozniak AS, Hansell DA (2017). Distribution of transparent exopolymer particles (TEP) across an organic carbon gradient in the western North Atlantic Ocean. Mar Chem.

[CR29] Jiang Q, Chiew YC (1994). Determination of ν- exponent for soluble polymeric monolayers at an air-water interface. Macromolecules.

[CR30] Joos P, Van Uffelen M, Serrien G (1992). Surface relaxation in spread insoluble monolayers of cholesterol and dipalmitoyl lecithin. J Coll Interf Sci.

[CR31] Kato T, Iriyama K, Araki T (1992). The time of observation of π-A isotherms. III. Studies on the morphology of arachidic acid monolayers, observed by transmission electron microscopy of replica samples of one-layer Langmuir-Blodgett films using plasma-polymerization. Thin Solid Films.

[CR32] Kragel J, Stortini AM, Degli-Innocenti N, Loglio G, Cini R, Miller R (1995). Dynamic interfacial properties of marine microlayers. Colloids and surfaces A: Physicochem Eng ASp.

[CR33] Liss PS, Riley JP, Skirrow G (1975). Chemistry of the sea surface microlayer. Chemical Oceanography.

[CR34] Maestro A, Guzman E, Ortega F, Rubio RG (2014). Contact angle of micro-and nanoparticles at fluid interfaces. Curr Opin Colloid & Interfaces.

[CR35] Maestro A, Santini E, Zabiegaj D, Llamas S, Ravera F, Liggieri L, Ortega F, Rubio RG, Guzman E (2015). Particle and particle-surfactant mixtures at fluid interfaces: assembly, morphology, and rheological description. Adv Condens Matter Phys.

[CR36] Mann EK, Lee LT, Henon S, Langevin D, Meunier J (1993). Polymer-surfactant films at the air-water interface. 1. Surface pressure, ellipsometry, and microscopic studies. Macromolecules.

[CR37] Mazurek AZ, Pogorzelski SJ (2012). Elastic properties of natural sea surface films incorporated with solid dust particles: model Baltic Sea studies. Int J Oceanogr.

[CR38] Mazurek AZ, Pogorzelski SJ, Kogut AD (2006). A novel approach for structure quantification of fatty acids on rain water. Atmos Environm.

[CR39] Mazurek AZ, Pogorzelski SJ, Kogut AD (2006). Structural characterization of fatty acid films on rain water: a scaling approach using physical attributes. Polish J Environ Stud.

[CR40] Mazurek AZ, Pogorzelski SJ, Boniewicz-Szmyt K (2008). Evolution of natural sea surface film structure as a tool for organic matter dynamics tracing. J Mar Systems.

[CR41] Meglen R (1992). Examining large databases: a chemometric approach using principle component analysis. Mar Chem.

[CR42] Miranda PB, Du Q, Shen YR (1998). Interaction of water with fatty acid Langmuir film. Chem Phys Lett.

[CR43] Periasamy V (2012). Mechanical and thermodynamic properties of chlorophyll-a surfactants at the air-water interface. Advan Mater Res.

[CR44] Pogorzelski SJ (1992). Isotherms of natural sea surface films: a novel device for sampling and properties studies. Rev Sci Instrum.

[CR45] Pogorzelski SJ (1996). Application of 2D polymer film scaling theory to natural sea surface films. Colloids and Surfaces A: Physicochem Eng ASp.

[CR46] Pogorzelski SJ (2001). Structural and thermodynamic characteristics of natural marine films derived from force-area studies. Colloids and surfaces A: Physicochem Eng ASp.

[CR47] Pogorzelski SJ, Kogut AD (2001). Static and dynamic properties of surfactant films on natural waters. Oceanologia.

[CR48] Pogorzelski SJ, Kogut AD (2001). Kinetics of marine surfactant adsorption at an air/water interface. Baltic Sea studies. Oceanologia.

[CR49] Pogorzelski SJ, Kogut AD (2003). Structural and thermodynamic signatures of marine microlayer films. J Sea Res.

[CR50] Pogorzelski SJ, Kogut AD (2003). Adsorptive properties of natural water surfactant films. Dead Vistula catchment water studies. Ocenologia.

[CR51] Pogorzelski SJ, Stortini AM, Loglio G (1994). Natural surface film studies in shallow coastal waters of the Baltic and Mediterranean Seas. Cont Shelf Res.

[CR52] Pogorzelski SJ, Kogut AD, Mazurek AZ (2006). Surface rheology parameters of source-specific surfactant films as indicators of organic matter dynamics. Hydrobiologia.

[CR53] Rosenholm JB, Ihalainen P, Peltonen J (2003). Thermodynamic characterization of Langmuir monolayers of thiolipids: a conceptual analysis. Colloids and surfaces A: Physicochem Eng ASp.

[CR54] Serrien G, Geeraerts G, Ghosh L, Joos P (1992). Dynamic surface properties of adsorbed protein solutions: BSA, casein and buttermilk. Colloids and Surfaces.

[CR55] Sieburth JM, Liss PS, Slinn WGN (1983). Microbiological and organic-chemical processes in the surface and mixed layers. Air-sea exchange of gases and particles.

[CR56] Song YK, Hong SH, Jang M, Kang JH, Kwon OY, Han GM, Shim WJ (2014). Large accumulation of micro-sized synthetic polymer particles in the sea surface microlayer. Environ Sci Technol.

[CR57] Song YK, Hong SH, Jang M, Han GM, Shim WJ (2015). Occurrence and distribution of microplastics in the sea surface microlayer in Jinhae Bay, South Korea. Arch Environ Contam Toxicol.

[CR58] Stolle C, Labrenz M, Meeske C, Jurgens K (2011). The bacterioneuston community structure of the southern Baltic Sea and its dependence on meteorological conditions. Appl and Environm Microbiol.

[CR59] Taylor JD, Cottingham SD, Billinge J, Cunliffe M (2014). Seasonal microbial community dynamics correlate with phytoplankton-derived polysaccharides in surface coastal waters. ISME J.

[CR60] Thornton DCO (2014). Dissolved organic matter (DOM) release by phytoplankton in the contemporary and future ocean. Eur J Phycol.

[CR61] Thornton DCO, Brooks SD, Chen J (2016). Protein and carbohydrate exopolymer particles in the sea surface microlayer (SML). Front Mar Sci.

[CR62] Verdugo P, Alldredge AL, Azam F, Kirchman DL, Passow U, Santschi PH (2004). The oceanic gel phase: a bridge in the DOM-POM continuum. Mar Chem.

[CR63] Woodcock LV (2016). Thermodynamics of criticality: percolation, loci, mesophases and critical dividing line in binary-liquid and liquid-gas equilibria. J Modern Phys.

[CR64] Wurl O, Ekau W, Landing WM, Zappa CJ (2017). Sea surface microlayer in a changing ocean—a perspective. Elem Sci Anth.

[CR65] Zhang Z, Liu L, Liu C, Cai W (2003). Studies on the sea surface microlayer. II The layer of sudden change of physical and chemical properties. J Colloid Interf Sci.

